# Impacts of Sex Differences in Pulse Pressure among Patients with Chronic Kidney Disease

**DOI:** 10.3390/jpm9040052

**Published:** 2019-12-09

**Authors:** Hiroshi Kataoka, Yukako Sawara, Keiko Kawachi, Shun Manabe, Toshio Mochizuki, Kosaku Nitta

**Affiliations:** 1 Department of Nephrology, Tokyo Women’s Medical University, 8-1 Kawada-cho, Shinjuku-ku, Tokyo 162-8666, Japan; 2 Department of Nephrology, Clinical Research Division for Polycystic Kidney Disease, Tokyo Women’s Medical University, Tokyo 162-866, Japan

**Keywords:** sex differences, pulse pressure, chronic kidney disease, visceral fat tissue, anemia, abdominal obesity

## Abstract

*Introduction*: Though disease-related differences between the sexes have increasingly attracted attention, the renal impact of pulse pressure (PP) in patients with chronic kidney disease (CKD) has never been investigated comprehensively in relation to differences associated with sex. We aimed to examine sex differences in PP as a related factor of CKD progression from the perspective of atherosclerosis. *Methods*: A total of 156 patients with CKD matched according to age and estimated glomerular filtration rate (eGFR) were separated into sex-based cohorts. Multivariate Cox proportional hazards analyses were performed to identify factors associated with renal outcomes. Kaplan–Meier analyses were performed to assess disease progression, which was defined as a ≥50% estimated glomerular filtration rate (eGFR) decline or end-stage renal disease. *Results*: The mean age of the study participants was 58.9 ± 13.1 years, and the median follow-up period was 114.0 months. A multivariate Cox regression analysis showed that PP was significantly associated with disease progression among the entire cohort (*p* = 0.007). In the sex-based sub-cohort analyses, PP was significantly associated with disease progression in men (*p* = 0.0004) but not in women. Among the entire cohort, PP was correlated positively with age (*p* = 0.03) and negatively with high-density lipoprotein-cholesterol (HDL-C) level (*p* = 0.003). PP was significantly correlated with visceral fat area (VFA) (*p* = 0.04) and hemoglobin level (*p* = 0.04) in men and with HDL-C level (*p* = 0.003) in women. *Conclusion*: A high PP is a significant related factor of CKD progression, especially in men, in whom it is significantly associated with greater VFA and lower hemoglobin level.

## 1. Introduction

Chronic kidney disease (CKD) is multifactorial, and its progression is often associated with a variety of risk factors linked to atherosclerosis, including hypertension, diabetes mellitus, dyslipidemia, obesity, and metabolic syndrome. Although hypertension is a major risk factor for CKD progression [1,2,3], recent clinical trials have failed to show the benefits of lower systolic blood pressure (SBP) in delaying disease progression [4,5,6]. A higher pulse pressure (PP), defined as the SBP minus the diastolic blood pressure (DBP), frequently reflects the arterial stiffness mediated by atherosclerosis [7]. A higher PP is often observed in older people and in patients with CKD. Given that PP measurements are easily obtained in clinical settings without special instruments, it is a valuable marker in CKD [8,9,10,11,12,13].

Recently, disease-related differences between the sexes have increasingly attracted attention. The risk factors associated with CKD progression have different effects according to sex [14], and being female seems to provide protection against development and progression of CKD [15,16]. Men show substantially higher rates of CKD and end-stage renal disease (ESRD) than women [17,18,19], which further suggests that men and women differ with respect to the pathogenesis and the clinical prognosis of CKD. Interestingly, older women have higher PP than men [20,21,22]. Nevertheless, the difference between the sexes in relation to PP as a progression-related factor in patients with CKD has never been elucidated in detail. Based on previous studies that demonstrated a lower risk of CKD progression [17,18,19] and a higher rate of high PP [20,21,22] in women, we hypothesized that the role of PP in CKD progression differs according to sex. Therefore, we aimed to examine sex differences in PP as a related factor of CKD progression from the perspective of atherosclerosis.

## 2. Materials and Methods

### 2.1. Study Population

**Figure 1:**
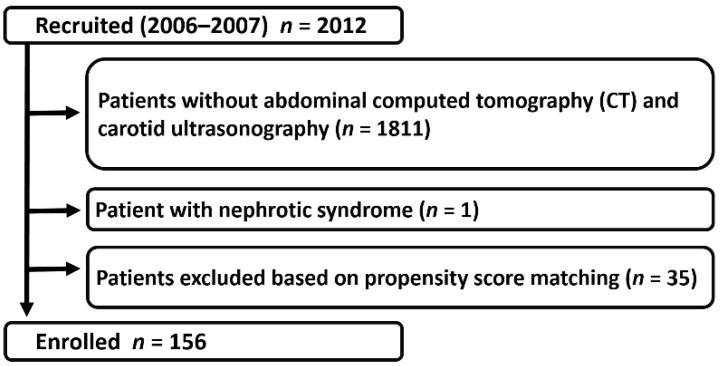
The patient selection flowchart is shown. Among 2012 screened patients, 1811 patients without abdominal computed tomography and carotid ultrasonography data, 1 patient with nephrotic syndrome, and 35 patients who were unmatched in the propensity score-matching analysis were excluded from the study. The remaining 156 patients were enrolled.

We screened 2012 CKD outpatients who visited the Kidney Center at Tokyo Women’s Medical University Hospital, Japan between August 2006 and August 2007. Among these, 201 patients without nephrotic syndrome underwent abdominal computed tomography (CT) and carotid ultrasonography. To better assess sex differences in PP as a related factor of CKD progression without the effects of age and kidney function, we fitted propensity score-matched models that included potential modifying variables, namely, age and estimated glomerular filtration rate (eGFR). After excluding patients who could not be matched, 156 patients were ultimately enrolled in the present study (Figure 1). CKD was diagnosed according to previously described criteria [23].

### 2.2. Covariate Assessments

During a regular outpatient clinic visit, anthropometric and physical examinations, including blood pressure (BP), body height, body weight, visceral fat area (VFA), subcutaneous fat area (SFA), and maximum carotid intima-media thickness (IMT) measurements, were conducted. BP was measured in triplicate using a mercury sphygmomanometer; the average value was used in analyses. The VFA and the SFA were measured using CT, and the IMT was measured using carotid ultrasonography. Details of the measurement techniques such as abdominal CT examination and carotid ultrasonography are provided in the Supplementary Materials.

All of the biochemical analyses were performed on samples obtained after overnight fasts. The serum creatinine levels were measured enzymatically. The eGFR for Japanese patients was calculated using a previously described formula [24]. The urinary albumin levels were measured using latex agglutination tests [25] and are expressed as the urine albumin-to-creatinine ratio (UACR). The impacts of concomitant drug use (antihypertensive drugs and diuretics, and drugs for the treatment of hyperuricemia, dyslipidemia, and diabetes mellitus) and comorbidities at entry (defined below) were also assessed.

Data on the following baseline parameters were collected: age, sex, SBP, DBP, mean BP (MBP), PP, body mass index (BMI), VFA, IMT, eGFR, UACR, and the levels of hemoglobin, serum albumin, uric acid (UA), low-density lipoprotein-cholesterol (LDL-C), high-density lipoprotein-cholesterol (HDL-C), triglyceride (TG), and high-sensitivity C-reactive protein (hs-CRP). PP was estimated as the difference between the SBP and the DBP. The participants were followed up until July 2016.

### 2.3. Definitions of the Comorbidities and Primary Causes of Chronic Kidney Disease

Hypertension was defined as an SBP ≥140 mmHg, DBP ≥90 mmHg, or taking an antihypertensive agent. Hyperuricemia was defined as a serum UA level ≥7.0 mg/dL or taking an antihyperuricemic agent. Diabetes mellitus was defined as glycated hemoglobin level ≥6.5%, diagnosis of diabetes mellitus, or intake of an antidiabetic agent. Hypertriglyceridemia was defined as a serum TG level ≥150 mg/dL or intake of an oral lipid-lowering agent. Hypercholesterolemia was defined as serum total cholesterol level ≥220 mg/dL, serum LDL-C level ≥140 mg/dL, or intake of an oral lipid-lowering agent. Low HDL-C was defined as a serum HDL-C level ≤40 mg/dL for men and ≤50 mg/dL for women. Diabetic kidney disease, chronic glomerulonephritis, and nephrosclerosis were diagnosed either from biopsies or clinically by the doctor in charge.

### 2.4. Study End Point

The study’s end point was kidney disease progression, which was defined as a ≥50% decline in eGFR [26] from baseline (≥50% eGFR decline) or the development of ESRD requiring dialysis.

### 2.5. Statistical Analysis

Continuous variables are expressed as means and standard deviations (SDs), and categorical variables are expressed as percentages, unless otherwise stated. Outcomes were compared using the unpaired *t*-test, chi-squared test, or Fisher’s exact test, as appropriate. Univariate and multivariate linear regression analyses were performed to determine the factors associated with the baseline PP level. Univariate and multivariate Cox proportional hazards analyses were performed to determine the variables associated with renal outcomes. Variables with *p* < 0.1 in the univariate analyses, as well as age, sex, and the eGFR, were included in the multivariate analyses. Subgroup analyses according to sex-based sub-cohorts (matched on age and eGFR) were performed. The caliper-matching method in the propensity score matching model used a maximum tolerance level of 0.2. *p*-values < 0.05 were considered statistically significant. All statistical analyses were performed using JMP Pro software, Windows v14.1.0 (SAS Institute, Cary, NC, USA).

## 3. Results

### 3.1. Patient Characteristics

Table 1 presents the baseline characteristics according to sex. The 156 participants comprised 78 men and 78 women, with a mean age at baseline of 58.9 ± 13.1 years (range: 24–84 years). The median follow-up duration was 114.0 months (interquartile range: 69.0–115.6 months), and 34 patients (20 men and 14 women) showed disease progression (i.e., a ≥50% eGFR decline or development of ESRD) during the follow-up period.

### 3.2. Pulse Pressure as a Progression-Related Factor in Patients with Chronic Kidney Disease

The results of the univariate and the multivariate Cox regression analyses are provided in Table 2, Table 3 and Table 4. The multivariate Cox regression analysis revealed that an increased PP was an independent risk factor for CKD progression among the entire cohort. The sex-based sub-cohort analyses revealed that a high PP was significantly associated with disease progression in men but not in women.

The multivariate Cox regression analyses also showed that, among the entire study cohort, disease progression was significantly associated with a 10 mL/min/1.73 m^2^ increase in eGFR [hazard ratio (HR): 0.49, 95% confidence interval (CI): 0.36–0.65; *p* < 0.0001], a 1 g/dL increase in serum albumin level (HR: 0.10, 95% CI: 0.03–0.32; *p* < 0.0001), age (HR: 0.92, 95% CI: 0.88–0.83; *p* = 0.0004), a 10 mmHg increase in PP (HR: 3.94, 95% CI: 1.32–10.03; *p* = 0.0070), and a 10 cm^2^ increase in VFA (HR: 1.08, 95% CI: 1.02–1.15; *p* = 0.0088) (Table 2).

Among men, disease progression was significantly associated with a 10 mL/min/1.73 m^2^ increase in eGFR (HR: 0.28, 95% CI: 0.15–0.51; *p* < 0.0001), a 1 g/dL increase in serum albumin level (HR: 0.02, 95% CI: 0.00–0.15; *p* < 0.0001), age (HR: 0.87, 95% CI: 0.81–0.94; *p* < 0.0001), and a 10 mmHg increase in PP (HR: 8.35, 95% CI: 1.32–24.53; *p* = 0.0004) (Table 3). Among women, disease progression was significantly associated with a 10 mL/min/1.73 m^2^ increase in eGFR (HR: 0.46, 95% CI: 0.24–0.77; *p* = 0.0027) and diabetes mellitus (HR: 4.49, 95% CI: 1.03–21.24; *p* = 0.0453) (Table 4).

### 3.3. Correlations between Pulse Pressure and Other Parameters

**Figure 2:**
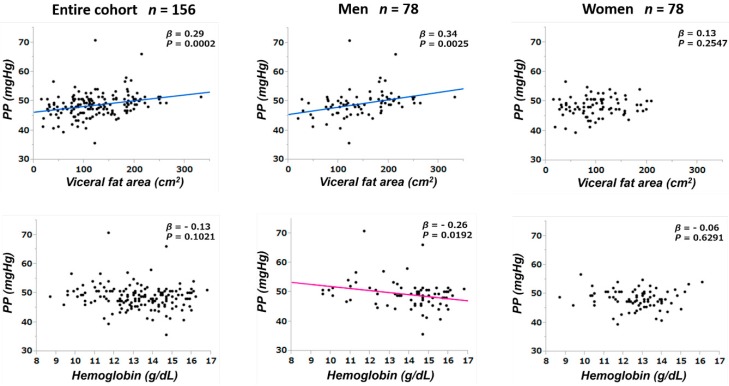
Relationships between the PP level and the visceral fat area (upper side) and the hemoglobin level (lower side) are shown for the entire cohort and for male and female sub-cohorts. PP, pulse pressure; *β*, standardized partial regression coefficient; *p*, calculated probability.

**Figure 3:**
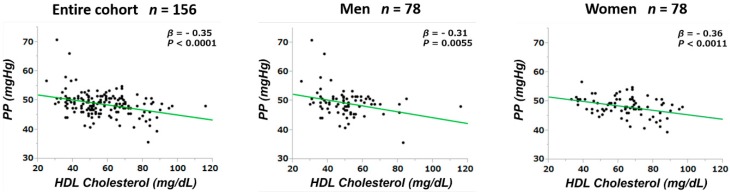
Relationships between the PP level and the high-density lipoprotein-cholesterol level are shown for the entire cohort and for male and female sub-cohorts. PP, pulse pressure; HDL, high-density lipoprotein; *β*, standardized partial regression coefficient; *p*, calculated probability.

Since PP values may be affected by confounders, baseline PP values were tested for correlations with clinical and laboratory parameters at baseline (Table 5 and Table 6, Figure 2 and Figure 3). The multivariate linear regression analyses showed that, among the entire population, PP was correlated with hemoglobin and serum HDL-C levels. The sex-based sub-cohort analyses revealed that PP was correlated with VFA and hemoglobin level in men and with HDL-C level in women.

The univariate analyses (Table 5) showed that, among the entire study cohort, PP was correlated significantly with age (*β* = 0.25; *p* = 0.0018), SBP (*β* = 0.62; *p* < 0.0001), MBP (*β* = 0.33; *p* < 0.0001), VFA (Figure 2, upper row: *β* = 0.29; *p* = 0.0002), IMT (*β* = 0.17; *p* = 0.0314), HDL-C (Figure 3: *β* = −0.35; *p* < 0.0001), hs-CRP (*β* = 0.22; *p* = 0.0057), and U-Prot (*β* = 0.31; *p* < 0.0001). In men, PP was correlated significantly with age (*β* = 0.25; *p* = 0.0251), SBP (*β* = 0.57; *p* < 0.0001), VFA (*β* = 0.34; *p* = 0.0025), hemoglobin (Figure 2, lower row: *β* = −0.26; *p* = 0.0192), HDL-C (*β* = −0.31; *p* = 0.0055), hs-CRP (*β* = 0.26; *p* = 0.0199), and U-Prot (*β* = 0.38; *p* = 0.0006) (Table 6). In women, PP was correlated significantly with age (*β* = 0.24; *p* = 0.0307), SBP (*β* = 0.69; *p* < 0.0001), DBP (*β* = 0.34; *p* = 0.0020), MBP (*β* = 0.49; *p* < 0.0001), and HDL-C (*β* = −0.36; *p* = 0.0011).

The multivariate correlational analyses showed that, among the entire study cohort, age and HDL-C were correlated significantly with PP (*β* = 0.19; *p* = 0.0259 and *β* = −0.25; *p* = 0.0030, respectively). The sex-based sub-cohort analyses showed that PP was correlated significantly with the VFA (*β* = 0.24; *p* = 0.0428) and hemoglobin (*β* = −0.30; *p* = 0.0395) in men and with HDL-C (*β* = −0.33; *p* = 0.0030) in women.

## 4. Discussion

We created sex-stratified cohorts that were matched for age and renal function and examined the CKD progression-related factors in each cohort. The study results demonstrate that there are differences between men and women in the related factors of CKD progression and that a high PP has greater significance in men than in women in terms of renal outcomes. These results add to the accumulating evidence that sex differences exist in patients with CKD in terms of incidence and prevalence of CKD, transition to ESRD, and mortality risk in patients undergoing renal replacement therapy [27]. Moreover, sex differences in atherosclerotic factors, namely, hypertension [28,29], diabetes mellitus [17], and BMI [30], have been described in relation to CKD progression.

A high PP is associated with an increase in the IMT in patients with hypertension [7] and may reflect arterial stiffness, which is associated with a process mediated by atherosclerosis. Atherosclerosis is common among patients with CKD, with atherosclerotic plaques characterized by higher levels of calcification [31] and high inflammatory marker levels [32]. The kidney is a high-blood-flow, low-resistance organ [33] that is vulnerable to hemodynamic changes. PP elevations caused by aortic stiffness as a consequence of increasing atherosclerosis with age can result in the transmission of a higher pressure and flow pulsatility in the kidney’s microvasculature, causing kidney damage [8,9,10,11,12,13]. Recent research into the influence of PP on renal outcomes suggests that PP is associated with CKD progression at all stages of the disease [8,10,13]. As with some atherosclerotic factors, PP appears to show sex differences with respect to CKD progression, as shown in the present study.

In the present study, PP at baseline was associated with CKD progression in men but not in women. The effect of sex on PP varies with age; PP is lower in women than in men during early adulthood but increases with age in women [21], resulting in a higher PP in women than in men at >60 years old [22]. These findings suggest that the mechanisms underlying a high PP in aging women differ from those in men. The presence of estrogen is thought to protect women against hypertension and kidney injury [34], and postmenopausal women lose this protection. The protection conferred on women before menopause may be related to the present finding that PP was not associated with CKD progression in women.

Additionally, in the present study, PP was correlated with VFA in men but not in women. High VFA is a component of metabolic syndrome and is considered to be an arteriosclerotic factor. When considering arteriosclerosis, it is impossible to disregard metabolic syndrome. Accumulating evidence suggests that patients with higher levels of body fat or metabolic syndrome have higher PP [35,36,37]; however, little is known about sex differences in the mechanisms underlying the development of high PP. Men and women display obvious differences in relation to body fat distribution [38]. Men are more likely to store visceral fat tissue, whereas women are more likely to develop subcutaneous fat tissue. Subcutaneous fat tissue differs from visceral fat tissue both anatomically and functionally, and visceral fat tissue may be more strongly correlated with metabolic cardiovascular risk factors than is subcutaneous fat tissue [39,40]. The amount of visceral fat tissue is higher in men compared to that in premenopausal women [41]. However, menopause and the associated decline in estrogen levels are associated with an increase in the amount of visceral fat tissue and adipocyte hypertrophy [42]; consequently, features associated with metabolic syndrome generally emerge in postmenopausal women [43,44]. The various vasoprotective effects of estrogen, including vasodilation, lipid profile improvements, and anti-inflammatory properties [45], may play significant roles in women with CKD, but these advantageous effects decline or are nonexistent in hyperglycemic states [46,47]. Indeed, the Cox proportional hazards showed a significant association between diabetes mellitus and CKD progression in women. Moreover, sex differences exist in the relationship between visceral fat and PP. For example, Shaikh et al. [48] reported a significant positive correlation between adiposity and PP in boys but not in girls, and sex hormone differences were thought to be the likely reason underlying these observations. Hence, there are sex differences in the relationships among PP, CKD progression, and visceral fat.

In the univariate analyses, PP was correlated significantly with the HDL-C level in both men and women. However, in the multivariate correlational analyses, PP was correlated significantly with HDL-C in women but not in men. Although the serum HDL-C levels tend to be higher in women than in men, it tends to decline in women as they age because of reductions in estrogen activity [49]. In addition, increases in the amount of visceral fat tissue as a consequence of menopause accelerate serum HDL-C level reductions in women. HDL-C is one of the most familiar metabolic syndrome components in women, and a low HDL-C level is strongly associated with the risk of mortality in older women [50]. Our results may mirror the characteristic serum HDL-C profile in women.

Additionally, the multivariate correlational analyses showed that PP was correlated negatively to the hemoglobin concentration in men but not in women. The effects of anemia on PP have been reported [51,52]; however, to the best of our knowledge, there are no reports on the effects of sex on PP in the context of anemia. Although the current clinical practice guidelines for the management of renal anemia [53] do not set any targets according to sex, men essentially have higher hemoglobin levels than do women [54]. The present study results suggest that, in the context of the guidelines for the management of renal anemia, PP may be more highly associated with anemia in men than in women.

As described above, PP is affected by a variety of factors that differ according to sex. Arteriosclerotic factors, including visceral fat, HDL-C, and PP, seem to be influenced by sex hormones that protect against arteriosclerosis and CKD progression. This may explain why we found that PP was not related to renal prognosis in women. However, our cohort was relatively small, and we cannot completely rule out PP as a related factor of renal prognosis in women. Indeed, Peralta et al. [13] reported that a high PP exacerbated kidney function decline with no significant interactions in relation to sex. However, the characteristics of the study cohort differed between our study and the study by Peralta et al. [13]. The mean BMI of the women in the study by Peralta et al. [13] was higher than that in the present study (28 ± 6 vs. 23.5 ± 4.4 kg/m^2^), which suggests that the protective effects of sex hormones may have been reduced. In an era in which personalized medicine has become expected [55], the finding that PP is of greater significance for men in relation to renal outcomes could be useful in clinical settings. Indeed, it may be necessary to seek more specific treatments for men who are likely to have poor renal prognosis.

The present study has several limitations. First, since all of the subjects were Japanese patients with CKD, the association between PP and renal outcomes may not be generalizable to other populations. Second, the impact of subsequent PP changes on outcomes was difficult to demonstrate because only baseline laboratory data were used in the analyses. Third, a potential selection bias was unavoidable because the patients voluntarily enrolled to participate in this study. Fourth, information about the menopausal status of the patients was not available in the present study. Fifth, the serum creatinine level was based on a single assessment at baseline, which may have been influenced by existing comorbidities at the time of the assessment. Sixth, information about the history of cardiovascular disease in patients was not available in the present study, but we cannot deny the possibility that cardiovascular disease had some influence on the renal outcome and pulse pressure. The strengths of the present study include its well-characterized population of Japanese patients with CKD who were treated by nephrologists at a single center using standard CKD care guidelines and the detailed analyses that were designed to disaggregate the data based on sex, which is significant for achieving patient-centered medicine [56,57].

## 5. Conclusions

The present study showed the existence of sex differences in the related factors of CKD progression and that a higher PP was a risk factor for CKD progression, particularly in men. In male patients with CKD, a high PP was associated with increased age, VFA, anemia, and proteinuria. Effectively treating the factors associated with a higher PP in men, including abdominal obesity or anemia, may suppress the progression of CKD. Thus, the present results suggest that clinicians should focus on sex-specific risk factors when treating patients with CKD.

## Figures and Tables

**Table 1:** Patient characteristics according to sex (*n* = 156).

Variables	Entire Cohort	Men	Female	*p*-Value
	*n* = 156	*n* = 78	*n* = 78	
*Clinical Findings*				
Age (years)	58.9 ± 13.1	59.2 ± 13.5	58.5 ± 12.8	0.7523
Gender (Men; %)	50.0	100.0	0.0	<0.0001
SBP (mmHg)	125.3 ± 7.9	126.8 ± 7.9	123.9 ± 7.8	0.0226
DBP (mmHg)	76.7 ± 6.3	77.6 ± 6.5	75.7 ± 6.0	0.0643
MBP (mmHg)	92.9 ± 6.6	94.0 ± 6.6	91.8 ± 6.5	0.0368
PP (mmHg)	48.7 ± 4.1	49.2 ± 4.7	48.2 ± 3.3	0.1187
BMI (kg/m^2^)	24.1 ± 4.1	24.6 ± 3.7	23.5 ± 4.4	0.0891
Visceral fat area (cm^2^)	123.5 ± 60.9	146.7 ± 63.3	100.4 ± 48.7	<0.0001
IMT (mm)	1.47 ± 0.77	1.83 ± 0.89	1.11 ± 0.38	<0.0001
*Laboratory Findings*				
Serum Albumin (g/dL)	4.20 ± 0.33	4.17 ± 0.37	4.22 ± 0.28	0.3515
Hemoglobin (g/dL)	13.3 ± 1.7	13.9 ± 1.8	12.7 ± 1.4	<0.0001
Serum Creatinine (mg/dL)	1.18 ± 0.84	1.33 ± 0.89	1.02 ± 0.76	0.0247
eGFR (mL/min/1.73m^2^)	56.4 ± 22.2	56.4 ± 22.3	56.4 ± 22.2	0.9991
Uric Acid (mg/dL)	5.84 ± 1.48	6.40 ± 1.31	5.28 ± 1.42	<0.0001
Triglyceride (mg/dL)	142.4 ± 69.9	160.9 ± 73.2	123.9 ± 61.5	0.0008
LDL Cholesterol (mg/dL)	115.4 ± 35.3	109.4 ± 32.0	121.5 ± 37.7	0.0327
HDL Cholesterol (mg/dL)	57.3 ± 16.5	50.8 ± 14.7	63.9 ± 15.7	<0.0001
Glucose (mg/dL)	106.5 ± 35.4	114.1 ± 46.7	98.9 ± 15.5	0.0072
Hemoglobin A1c (NGSP) (%)	6.05 ± 0.92	6.05 ± 0.85	6.05 ± 1.01	0.9804
Hs-CRP (ng/mL)	777.8 ± 1052.3	964.7 ± 1245.9	590.8 ± 778.5	0.0270
UACR (mg/g Cre)	303.8 ± 614.3	451.2 ± 779.7	156.4 ± 328.2	0.0025
U-Prot (g/day)	0.65 ± 1.23	0.93 ± 1.51	0.36 ± 0.76	0.0035
*Primary cause of CKD*				
Diabetic nephropathy (%)	6.4	7.7	5.1	0.7456
Chronic glomerulonephritis (%)	52.6	44.9	60.3	0.0543
Nephrosclerosis (%)	21.8	34.6	9.0	0.0001
Others (%)	19.2	12.8	25.6	0.0422
*Concomitant drugs*				
Antihypertensive agents (%)	68.6	76.9	60.3	0.0249
ARB and or ACEI	55.1	66.7	43.6	0.0038
Ca blockade	30.8	28.2	33.3	0.4878
Antihyperuricemic agents (%)	38.5	53.9	23.1	<0.0001
Antidiabetic agents (%)	13.5	15.4	11.5	0.4816
Corticosteroids (%)	15.4	16.7	14.1	0.6572
Immunosuppressants (%)	6.4	6.4	6.4	1.0000
Diuretics (%)	23.1	16.7	29.5	0.0574
*Comorbidities*				
Hypertension (%)	68.0	75.6	60.3	0.0395
Hyperuricemia (%)	48.7	66.7	30.8	<0.0001
Hypertriglyceridemia (%)	58.3	64.1	52.6	0.1439
Hypercholesterolemia (%)	58.3	55.1	61.5	0.4168
Low HDL cholesterol (%)	50.0	51.3	48.7	0.7488
Diabetes mellitus (%)	19.2	20.5	18.0	0.6845

Continuous variables are expressed as means and standard deviations. Categorical variables are expressed as percentages. Abbreviations: *n*, number; CKD, chronic kidney disease; SBP, systolic blood pressure; DBP, diastolic blood pressure; MBP, mean blood pressure; PP, pulse pressure; BMI, body mass index; IMT, maximum carotid intima-media thickness; eGFR, estimated glomerular filtration rate; LDL-C, low-density lipoprotein cholesterol; HDL-C, high-density lipoprotein cholesterol; TG, triglyceride; Hs-CRP, high sensitivity C-reactive protein; UACR, urine albumin-to-creatinine ratio; U-Prot, urinary protein excretion; ARB, angiotensin II receptor blocker; ACEI, angiotensin converting enzyme inhibitor.

**Table 2:** Results of the univariate and the multivariate analyses for the risk factors associated with disease progression (i.e., a ≥50% estimated glomerular filtration rate decline or end-stage renal disease) among the entire study population (*n* = 156).

Variables	Univariate Analysis		Multivariate Analysis	
	Hazard Ratio (95% CI)	*p*-Value	Hazard Ratio (95% CI)	*p*-Value
Age (1 year increase)	1.00 (0.98–1.03)	0.7421	0.92 (0.88–0.96)	0.0004
Men (vs. women)	1.69 (0.86–3.43)	0.1266	2.08 (0.54–8.40)	0.1391
SBP (10 mmHg increase)	1.80 (1.12–2.96)	0.0149	-	-
DBP (10 mmHg increase)	1.43 (0.81–2.58)	0.2220	-	-
MBP (10 mmHg increase)	1.64 (0.94–2.89)	0.0814	-	-
PP (10 mmHg increase)	2.56 (1.25–4.60)	0.0117	3.94 (1.32–10.03)	0.0070
Visceral fat area (10 cm^2^ increase)	1.09 (1.03–1.14)	0.0013	1.08 (1.02–1.15)	0.0088
IMT (1 mm increase)	1.42 (0.97–1.96)	0.0722	1.57 (0.85–2.72)	0.1125
eGFR (10 mL/min/1.73 m^2^ increase)	0.52 (0.41–0.63)	<0.0001	0.49 (0.36–0.65)	<0.0001
Hemoglobin (1 g/dL increase)	0.62 (0.52–0.75)	<0.0001	0.88 (0.67–1.15)	0.2966
Serum albumin (1 g/dL increase)	0.14 (0.07–0.31)	<0.0001	0.10 (0.03–0.32)	<0.0001
Hs-CRP (10 ng/mL increase)	1.00 (1.00–1.00)	0.0761	1.00 (1.00–1.00)	0.2528
U-Prot (g/day)	1.60 (1.38–1.81)	<0.0001	-	-
Hypertriglyceridemia (vs. no)	1.43 (0.71–3.04)	0.3234	-	-
Hypercholesterolemia (vs. no)	1.25 (0.63–2.61)	0.5274	-	-
Low HDL cholesterol (vs. no)	2.53 (1.25–5.55)	0.0096	1.30 (0.53–3.30)	0.5487
Hypertension (vs. no)	4.10 (1.62–13.81)	0.0017	1.05 (0.38–3.75)	0.9257
Hyperuricemia (vs. no)	6.44 (2.85–17.22)	<0.0001	1.04 (0.35–3.31)	0.9495
Diabetes mellitus (vs. no)	2.29 (1.07–4.60)	0.0332	2.05 (0.81–5.06)	0.1237

Variables with a *p*-value < 0.1 in the univariate model, as well as age, sex, PP, and the estimated glomerular filtration rate, were included in the multivariate model. Urine protein excretion was not included in the multivariate model given its causality. Abbreviations: ESRD, end-stage renal disease; *n*, number; CI, confidence interval; PP, pulse pressure; IMT, maximum carotid intima-media thickness; eGFR, estimated glomerular filtration rate; Hs-CRP, high sensitivity C-reactive protein; UACR, urine albumin-to-creatinine ratio; U-Prot, Urinary protein excretion; vs, versus.

**Table 3:** Results of the univariate and the multivariate analyses for the risk factors associated with disease progression (i.e., a ≥50% estimated glomerular filtration rate decline or end-stage renal disease) in men (*n* = 78).

Variables	Univariate Analysis		Multivariate Analysis	
	Hazard Ratio (95% CI)	*p*-Value	Hazard Ratio (95% CI)	*p*-Value
Age (1 year increase)	1.01 (0.97–1.05)	0.6684	0.87 (0.81–0.94)	<0.0001
SBP (10 mmHg increase)	2.64 (1.28–5.80)	0.0080	-	-
DBP (10 mmHg increase)	1.36 (0.62–3.13)	0.4486	-	-
MBP (10 mmHg increase)	1.89 (0.84–4.45)	0.1233	-	-
PP (10 mmHg increase)	2.61 (1.27–4.69)	0.0035	8.35 (2.4–24.53)	0.0004
Visceral fat area (10 cm^2^ increase)	1.06 (0.99–1.13)	0.1127	-	-
IMT (1 mm increase)	1.30 (0.81–1.93)	0.2609	-	-
eGFR (10 mL/min/1.73 m^2^ increase)	0.52 (0.39–0.67)	<0.0001	0.28 (0.15–0.51)	<0.0001
Hemoglobin (1 g/dL increase)	0.56 (0.44–0.71)	<0.0001	1.00 (0.67–1.48)	0.9933
Serum albumin (1 g/dL increase)	0.14 (0.06–0.32)	<0.0001	0.02 (0.00–0.15)	<0.0001
Hs-CRP (10 ng/mL increase)	1.00 (1.00–1.00)	0.0761	1.00 (1.00–1.00)	0.8530
U-Prot (g/day)	1.52 (1.29–1.76)	<0.0001	-	-
Hypertriglyceridemia (vs. no)	1.36 (0.53–4.18)	0.5449	-	-
Hypercholesterolemia (vs. no)	1.85 (0.74–5.22)	0.1931	-	-
Low HDL cholesterol (vs. no)	2.04 (0.82–5.76)	0.1286	-	-
Hypertension (vs. no)	7.05 (1.46–126.65)	0.0098	1.95 (0.23–16.22)	0.5041
Hyperuricemia (vs. no)	6.08 (1.75–38.27)	0.0025	0.37 (0.06–2.36)	0.3139
Diabetes mellitus (vs. no)	1.75 (0.62–4.37)	0.2704	-	-

**Table 4:** Results of the univariate and the multivariate analyses for the risk factors associated with disease progression (i.e., a ≥50% estimated glomerular filtration rate decline or end-stage renal disease) in women (*n* = 78).

Variables	Univariate Analysis		Multivariate Analysis	
	Hazard Ratio (95% CI)	*p*-Value	Hazard Ratio (95% CI)	*p*-Value
Age (1 year increase)	1.00 (0.96–1.04)	0.8692	0.98 (0.91–1.04)	0.4647
SBP (10 mmHg increase)	1.21 (0.62–2.40)	0.5731	-	-
DBP (10 mmHg increase)	1.39 (0.58–3.31)	0.4568	-	-
MBP (10 mmHg increase)	1.33 (0.59–2.98)	0.4917	-	-
PP (10 mmHg increase)	0.99 (0.21–4.99)	0.9930	-	-
Visceral fat area (10 cm^2^ increase)	1.16 (1.05–1.29)	0.0047	1.04 (0.93–1.17)	0.4602
IMT (1 mm increase)	1.34 (0.34–3.52)	0.6251	-	-
eGFR (10 mL/min/1.73 m^2^ increase)	0.50 (0.34–0.70)	<0.0001	0.93 (0.58–1.45)	0.0027
Hemoglobin (1 g/dL increase)	0.59 (0.43–0.82)	0.0019	0.93 (0.58–1.45)	0.7560
Serum albumin (1 g/dL increase)	0.32 (0.03–2.37)	0.2430	-	-
Hs-CRP (10 ng/mL increase)	1.00 (1.00–1.00)	0.8702	-	-
U-Prot (g/day)	1.54 (0.98–2.12)	0.0573	-	-
Hypertriglyceridemia (vs. no)	1.29 (0.45–3.93)	0.6322	-	
Hypercholesterolemia (vs. no)	0.78 (0.27–2.36)	0.6419	-	-
Low HDL cholesterol (vs. no)	3.23 (1.08–11.80)	0.0356	1.93 (0.48–9.09)	0.3595
Hypertension (vs. no)	2.71 (0.85–12.00)	0.0965	1.83 (0.51–8.66)	0.3708
Hyperuricemia (vs. no)	6.50 (2.17–23.71)	0.0007	1.17 (0.21–6.47)	0.8519
Diabetes mellitus (vs. no)	3.13 (0.96–9.08)	0.0574	4.49 (1.03–21.24)	0.0445

Variables with a *p*-value < 0.1 in the univariate model, as well as age, sex, PP, and the estimated glomerular filtration rate, were included in the multivariate model. Urine protein excretion was not included in the multivariate model given its causality. Abbreviations: ESRD, end-stage renal disease; *n*, number; CI, confidence interval; PP, pulse pressure; IMT, maximum carotid intima-media thickness; eGFR, estimated glomerular filtration rate; Hs-CRP, high sensitivity C-reactive protein; UACR, urine albumin-to-creatinine ratio; U-Prot, Urinary protein excretion; vs., versus.

**Table 5:** Results of the univariate and the multivariate linear regression analyses for the factors associated with baseline PP among the entire cohort (*n* = 156).

Variables	Univariate Analysis		Multivariate Analysis	
	*β*	*p*-Value	*β*	*p*-Value
Age (years)	0.25	0.0018	0.13	0.1405
	0.62	<0.0001	-	-
DBP (mmHg)	0.13	0.1068	-	-
MBP (mmHg)	0.33	<0.0001	-	-
Visceral fat area (cm^2^)	0.29	0.0002	0.14	0.1132
IMT(mm)	0.17	0.0314	0.01	0.9095
eGFR (mL/min/1.73 m^2^)	−0.07	0.3995	0.13	0.1531
Hemoglobin (g/dL)	−0.13	0.1021	−0.21	0.0304
Serum albumin (g/dL)	−0.06	0.4773	0.09	0.2961
Uric acid (mg/dL)	0.02	0.7743	-	-
Triglyceride (mg/dL)	0.06	0.4350	-	-
LDL cholesterol (mg/dL)	−0.10	0.2108	-	-
HDL cholesterol (mg/dL)	−0.35	<0.0001	−0.27	0.0018
Glucose (mg/dL)	0.06	0.4685	-	-
Hemoglobin A1c (NGSP) (%)	0.04	0.7030	-	-
Hs-CRP (ng/mL)	0.22	0.0057	0.08	0.0807
U-Prot (g/day)	0.31	<0.0001	-	-

**Table 6:** Results of the univariate and the multivariate linear regression analyses for the factors associated with baseline PP in men and in women.

Variables	Men (*n* = 78)				Women (*n* = 78)			
	Univariate		Multivariate		Univariate		Multivariate	
	*β*	*p*-Value	*β*	*p*-Value	*β*	*p*-Value	*β*	*p*-Value
Age (years)	0.25	0.0251	0.11	0.3789	0.24	0.0307	0.18	0.0914
SBP (mmHg)	0.57	<0.0001	-	-	0.69	<0.0001	-	-
DBP (mmHg)	−0.04	0.7587	-	-	0.34	0.0020	-	-
MBP (mmHg)	0.20	0.0725	-	-	0.49	<0.0001	-	-
Visceral fat area (cm^2^)	0.34	0.0025	0.24	0.0428	0.13	0.2547	-	-
IMT (mm)	0.13	0.2626	-	-	0.15	0.2002	-	-
eGFR (mL/min/1.73 m^2^)	−0.12	0.3155	0.19	0.1629	−0.00	0.9722	-	-
Hemoglobin (g/dL)	−0.26	0.0192	−0.30	0.0395	−0.06	0.6291	-	-
Serum albumin (g/dL)	−0.09	0.4331	-	-	0.03	0.7855	-	-
Uric acid (mg/dL)	0.01	0.9144	-	-	−0.08	0.4957	-	-
Triglyceride (mg/dL)	0.01	0.9024	-	-	0.06	0.6016	-	-
LDL cholesterol (mg/dL)	−0.21	0.0660	-	-	0.07	0.5549	-	-
HDL cholesterol (mg/dL)	−0.31	0.0055	−0.15	0.1886	−0.36	0.0011	−0.33	0.0030
Glucose (mg/dL)	0.01	0.8989	-	-	0.11	0.3476	-	-
Hemoglobin A1c (NGSP) (%)	−0.00	0.9966	-	-	0.09	0.5053	-	-
Hs-CRP (ng/mL)	0.26	0.0199	0.18	0.1007	0.06	0.5808	-	-
U-Prot (g/day)	0.38	0.0006	-	-	0.05	0.6895	-	-

Variables with a *p*-value < 0.1 in the univariate model, as well as age and the estimated glomerular filtration rate, were included in the multivariate model. Factors related to blood pressure and urine protein excretion were not included in the multivariate model given their causality. Abbreviations: n, number; *β*, standardized partial regression coefficient; *p*, calculated probability; PP, pulse pressure; SBP, systolic blood pressure; DBP, diastolic blood pressure; MBP, mean blood pressure; IMT, maximum carotid intima-media thickness; eGFR, estimated glomerular filtration rate; LDL-C, low-density lipoprotein cholesterol; HDL-C, high-density lipoprotein cholesterol; Hs-CRP, high sensitivity C-reactive protein; U-Prot, Urinary protein excretion.

## Supplementary Materials

The following are available online at https://www.mdpi.com/2075-4426/9/4/52/s1. The appendix provides further methodological details (abdominal CT examination and carotid ultrasonography) and references that support this work.
