# Supplementary material for: Impacts of Sex Differences in Pulse Pressure among Patients with Chronic Kidney Disease
Source: J Pers Med. 2019 Dec 9;9(4):52. doi: 10.3390/jpm9040052 (PMC6963663; doi:10.3390/jpm9040052)
Supplement: Supplementary file 1 [file jpm-09-00052-s001.pdf]

Supplementary Methods

Abdominal CT Examination

A multidetector-row CT examination was performed by using a GE LightSpeed. An index image was obtained before scanning, and the umbilicus to L4–5 level was identified as previously described [1, 2]. Horizontal images were obtained at 400 mA and 120 kVp with a scan time of 1.0 s. The range of CT values covered the optimal CT numbers for adipose tissue, i.e., from −150 to −40.14 Data were stored and analyzed with GE advantage workstation Ver.4.0.

Carotid Ultrasonography

An experienced examiner blinded to the patient data performed high-resolution duplex carotid ultrasonography with a 7.5-MHz duplex scanner (Aplio XG; Toshiba, Tokyo, Japan) as previously described [3, 4]. The common and internal carotid arteries were scanned cross-sectionally and longitudinally to estimate the presence and distribution of atherosclerotic plaques. The entire length of both common carotid arteries and both internal carotid arteries was scanned up to approximately 20 mm distal to the tip of the carotid bifurcation. IMT measurements were obtained to identify the thickest region of the arterial wall according to an international consensus report [5].

1 Sawara Y, Takei T, Uchida K, Tsuchiya K, Nitta K: Metabolic syndrome and anthropometric factors in Japanese patients with chronic kidney disease. Heart and vessels 2009;24:199-203.

2 Tamei N, Ogawa T, Ishida H, Ando Y, Nitta K: Relationship of high-molecular-weight adiponectin levels to visceral fat accumulation in hemodialysis patients. Intern Med 2010;49:299-305.

3 Ogawa T, Shimada M, Ishida H, Matsuda N, Fujiu A, Ando Y, Nitta K: Relation of stiffness parameter beta to carotid arteriosclerosis and silent cerebral infarction in patients on chronic hemodialysis. International urology and nephrology 2009;41:739-745.

4 Sato M, Ogawa T, Sugimoto H, Otsuka K, Nitta K: Relation of carotid intima-media thickness and silent cerebral infarction to cardiovascular events and all-cause mortality in chronic hemodialysis patients. Internal medicine (Tokyo, Japan) 2012;51:2111-2117.

5 de Bray JM, Baud JM, Delanoy P, Camuzat JP, Dehans V, Descamp-Le Chevoir J, Launay JR, Luizy F, Sentou Y, Cales P: Reproducibility in ultrasonic characterization of carotid plaques. Cerebrovascular diseases (Basel, Switzerland) 1998;8:273-277.
